# New insight into the role of SOCS family in immune regulation and autoimmune pathogenesis

**DOI:** 10.1016/j.jare.2025.05.020

**Published:** 2025-05-10

**Authors:** Shiyi Liu, Mingwei Wang, Liangjie Xu, Daihua Deng, Liwei Lu, Jie Tian, Dongmei Zhou, Ke Rui

**Affiliations:** a Department of Laboratory Medicine, Affiliated Hospital of Jiangsu University, Zhenjiang, China; b Department of Immunology, Jiangsu Key Laboratory of Laboratory Medicine, School of Medicine, Jiangsu University, Zhenjiang, China; c Department of Emergency, Affiliated People’s Hospital of Jiangsu University, Zhenjiang, China; d Department of Cardiology, Affiliated Hospital of Jiangsu University, Zhenjiang, China; e Department of Rheumatology, Mianyang Central Hospital, School of Medicine, University of Electronic Science and Technology of China, Mianyang, China; f Department of Pathology and Shenzhen Institute of Research and Innovation, The University of Hong Kong, Chongqing International Institute for Immunology, China; g Department of Rheumatology and Immunology, Affiliated Hospital of Xuzhou Medical University, Xuzhou, China

**Keywords:** Suppressor of cytokine signaling, Immune cell, Immunomodulation, Autoimmune disease

## Abstract

**Background:**

Suppressor of cytokine signaling (SOCS) proteins regulate signal transduction by interacting with cytokine receptors and signaling proteins and targeting associated proteins for degradation. Recent studies have demonstrated that the SOCS proteins serve as crucial inhibitors in cytokine signaling networks and play a pivotal role in both innate and adaptive immune responses.

**Aim of Review:**

In this review, we aim to discuss recent advancements in understanding the complex functions of SOCS proteins in various immune cells, as well as the effects of SOCS proteins in human health and diseases. Increasing evidence indicates that SOCS proteins are frequently dysregulated in developing autoimmune diseases, suggesting that therapeutic targeting of SOCS proteins could provide clinical benefit.

**Key Scientific Concepts of Review:**

This review provides a comprehensive understanding of SOCS proteins in immune regulation and autoimmune pathogenesis, it also highlights the role of SOCS-related mimetic peptides in immunotherapy.

## Highlights

• SOCS proteins regulate the differentiation and function of innate immune cells and lymphoid cells by modulating multiple signaling pathways.
• The expression of SOCS proteins can regulate the development of autoimmune diseases.
• SOCS-related mimetic peptides serve as potential therapeutic targets for the treatment of autoimmune disorders.

## Graphical abstract

**Figure:**
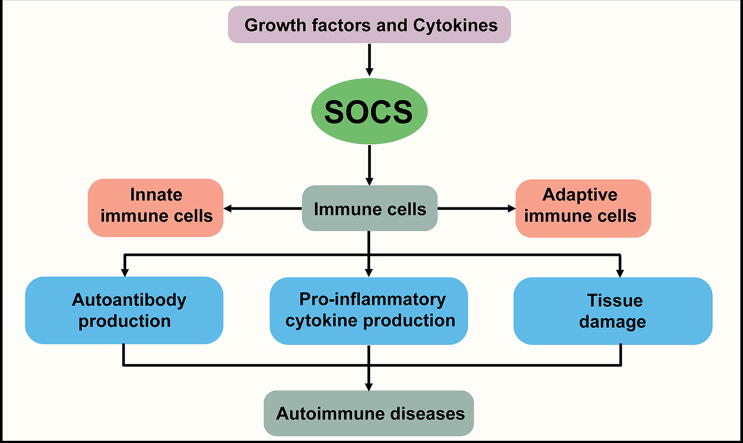

## Introduction

The eight members of the suppressor of cytokine signaling (SOCS) protein family, which includes cytokine-inducible SH2-containing protein (CIS, also known as CISH) and SOCS1-SOCS7, are negative regulatory proteins that have the ability to suppress cytokine signaling and modulate the immune cell response to cytokines[1,2]. Physiological stimulation of cytokines results in the process of janus kinase (JAK)-mediated tyrosine phosphorylation of signal transducer and activator of transcription (STAT), going through a series of processes that include dimerization, translocation into the nucleus, eventually regulating gene expression[3]. Meanwhile, SOCS accomplishes a negative feedback loop in the JAK/STAT signaling pathway: activated STATs stimulate the transcription of the SOCS gene, subsequently, SOCS proteins bind to the phosphorylated JAK and the JAK receptor, shutting down the pathways[4]. Nevertheless, in pathology conditions such as autoimmune diseases, autoantibodies or inflammatory factors (e.g. IL-6, TNF-α) result in the over-activation of JAK/STAT signaling, thereby leading to sustained phosphorylation of STAT3/STAT5, which promotes pro-inflammatory factors, such as IL-17, and exacerbates tissue damage[3]. This may be due to insufficient expression or aberrant function of SOCS proteins, resulting in negative feedback failure. Thus, SOCS proteins act as molecular rheostats, the functional integrity of which determines the disease process.

**Fig. 1:**
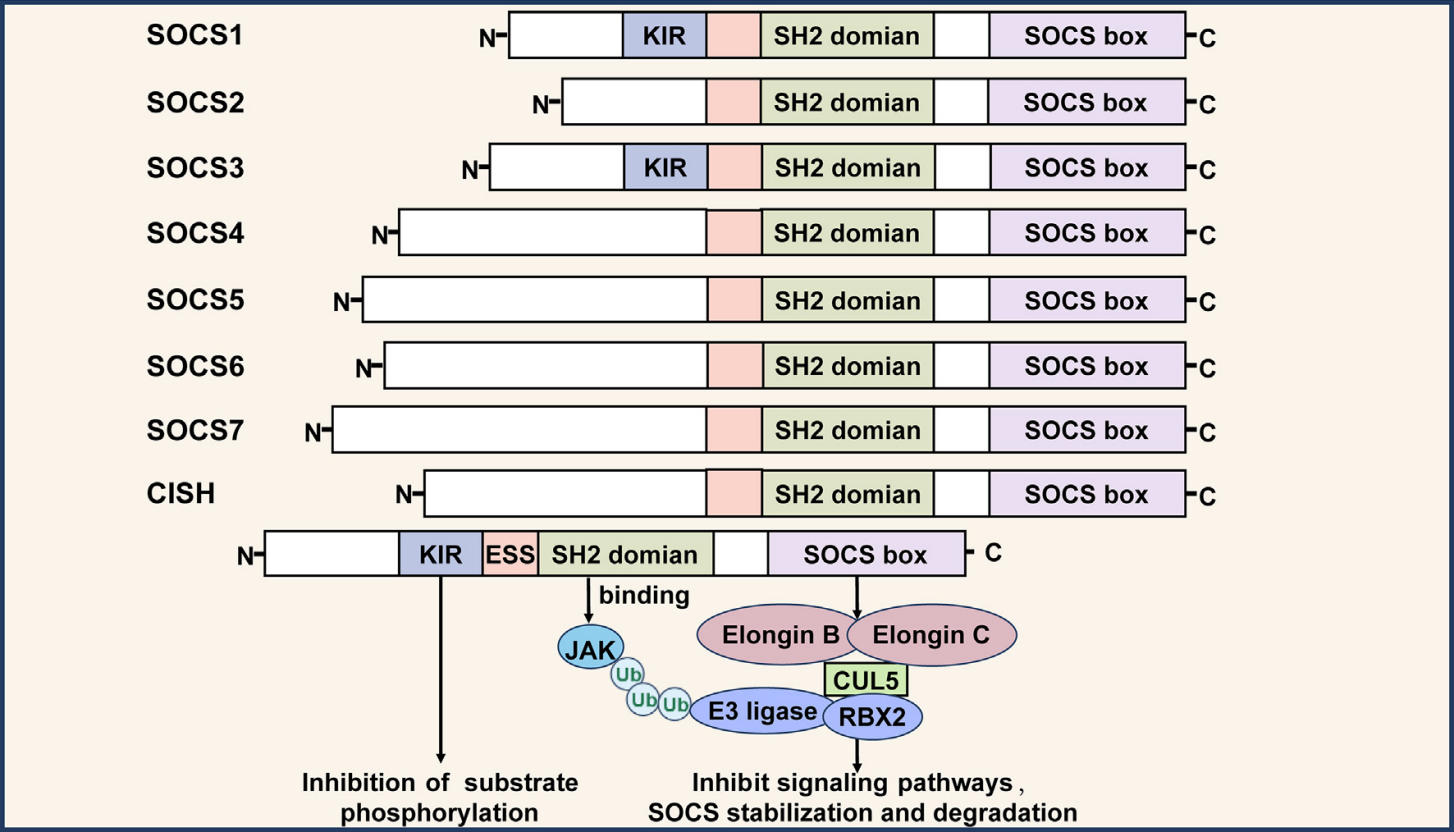
The structure and function of the SOCS family.

SOCS molecules exhibit structural similarity, comprising a low conservation N-terminal domain, an established core Src-homology 2 (SH2) domain, as well as an extremely conserved C-terminal domain identified as the SOCS box[5]. The N-terminus domain of SOCS molecules is diverse in length. SOCS4-SOCS7 show extended N-terminus domains than SOCS1-3[6]. SOCS1 and SOCS3 possess a kinase inhibitory region (KIR) upstream of the SH2 domain[7]. The SH2 domain recognizes and binds cognate phosphotyrosine motifs, specifying the objective for each SOCS/CISH molecule to perform its regulation[5]. Research indicates that the binding of SOCS1 to Tyr441 of interferon-gamma receptor 1 (IFNGR1) blocks signal transducers and STAT1 from accessing Tyr419, thereby preventing STAT1 activation[8]. Similarly, the SH2 domain of SOCS3 connects to the STAT4 binding site in the IL-12R-β2 subunit, reducing STAT4 activation. Furthermore, the SH2 domain of murine SOCS3 includes an unstructured PEST motif insertion, acting as an essential regulator of protein stability[9]. The N-terminus contains an SH2 substructural domain (ESS) that facilitates substrate interactions[10]. The SOCS box engages with ubiquitinating enzymes, including Elongin B, Elongin C, Cullin 5 (CUL5), Ring-box protein 2 (RBX2), along with an E2 ubiquitin transferase to establish the E3 ubiquitin ligase complex, which targets JAKs and cytokine receptors for degradation via the proteasome[10]. Additionally, SOCS3 protein expression is stabilized by the connection with Elongin C, but this complex is disrupted and proteasome-mediated degradation of SOCS3 is enhanced when SOCS3 box tyrosine residues are phosphorylated[11]. For instance, SOCS3 can be phosphorylated at Tyr204 and Tyr221 to prevent the SOCS3-Elongin C binding and to initiate SOCS3 proteasome degradation[11]. Similarly, the SOCS box of CISH degrading substrates requiring Elongin B and Elongin C. A study has shown that a CIS mutant deficient in recruitment of the Elongin B and Elongin C failed to suppress STAT5 activation[12] (Fig. 1).

The SH2 domain and the SOCS box domain at the C-terminus are the fundamental components of the SOCS molecule structure. Each member has a different length for the N-terminal domain. Notably, both SOCS1 and SOCS3 contain a kinase inhibitory region (KIR) domain preceding the SH2 domain. From SOCS4 to SOCS7, they have a longer N-terminal domain than SOCS1-3 and CISH. The SOCS box assembles an E3 ubiquitin ligase complex to mediate proteasomal degradation of JAKs and cytokine receptors.

## The mechanisms of SOCS protein

**Fig. 2:**
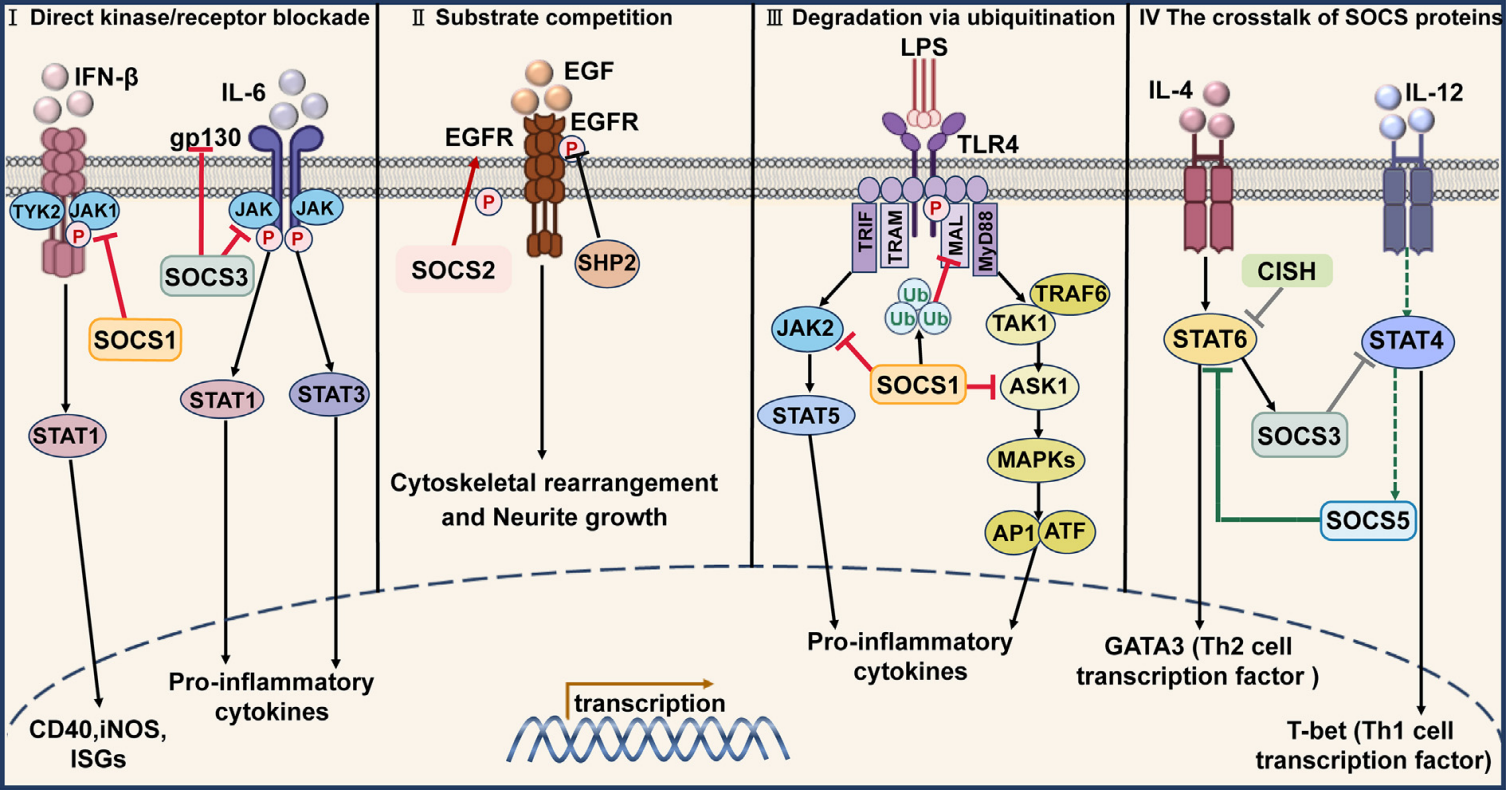
Overview of cytokine signaling pathways involved in SOCS proteins.

SOCS molecules function as typical adverse feedback inhibitors, activated by diverse cytokines, and attenuate cytokine-induced signaling pathways[13]. The growth factors and cytokines that induce SOCS are summarized in Table 1. While different SOCS proteins exhibit context-dependent roles, their inhibitory mechanisms converge on three conserved strategies: (Ⅰ) direct kinase/receptor blockade, (Ⅱ) substrate competition, and (Ⅲ) ubiquitination-mediated degradation[14] (Fig. 2).

**Table 1:** Factors that induce SOCS family proteins.

**SOCS proteins**	**Growth factors and Cytokines that induce SOCS**	**Ref.**
SOCS1	IL-2, IL-4, IL-6, IL-7, IL-10, G-CSF, GH, CNTF, CT1, EPO, IFN-α/β/γ, Insulin, LIF, prolactin, SCF, TNF-α, TSH, PRL	[[184], [185], [186], [187], [188], [189], [190], [191], [192], [193]]
SOCS2	IL-2, IL-6, GH, CNTF, EPO, GH, Insulin	[180,181]
SOCS3	IL-1, IL-2, IL-3, IL-4, IL-6, IL-9, IL-10, IL-11, IL-12, IL-22, IL-23, IL-27, CNTF, CT1, EPO, IFN-α/β/γ, Insulin, LIF, TNF-α, TSH, EGF, Leptin	[85,122,152,[180], [181], [182], [183], [184], [185]]
SOCS4	EGF	[180]
SOCS5	EGF	[180]
SOCS6	SCF, Insulin	[180,181]
SOCS7	Simvastain, Insulin	[139,180]
CISH	IL-2, IL-3, IL-6, IL-9, IL-10, CNTF, EGF, EPO, GH, GM-CSF, Leptin, TPO, TSLP, PRL	[[180], [181], [182], [183], [184], [185], [186], [187]]

G-CSF, Granulocyte Colony-Stimulating Factor; GH,Growth Hormone; CNTF, Ciliary Neurotrophic Factor; CT1, Cardiotrophin-1; EPO, erythropoietin; LIF, Leukemia Inhibitory Factor; SCF, Stem Cell Factor; TNF-α, Tumor Necrosis Factor-α; TSH, Thyroid Stimulating Hormone; PRL, prolactin; EGF, Epidermal Growth Factor; GM-CSF: Granulocyte-macrophage colony-stimulating factor; TPO, Thrombopoietin; TSLP, Thymic stromal lymphopoietin.

### SOCS1 protein

SOCS molecules can bind to the activation loop to directly eliminate JAK activity[15]. For instance, SOCS1 exclusively targets JAK through its KIR and SH2 domains by binding to the JAK groove to block phosphorylation[15]. Moreover, SOCS proteins block signaling pathways by directing related substrates for destruction with ubiquitination[16,17]. It has been found that SOCS1 interacts with phosphorylated MyD88 adapter-like protein (MAL), leading to the polyubiquitination and degradation of MAL[16].

### SOCS2 protein

SOCS can interfere with cytokine signaling by substrate competition[18]. In this regard, SOCS2 interacts with tyrosine-phosphorylated epidermal growth factor receptor (EGFR) and competes with Src tyrosine kinase (SHP2) for the binding site of EGFR, inhibiting phosphatase SHP2 dephosphorylation, thereby maintaining sustained EGFR activation and inducing neurite growth[18].

### SOCS3 protein

SOCS3 expression can be induced by IL-6, IL-12, IL-23, and granulocyte colony-stimulating factor (G-CSF) cytokine signaling. Unlike SOCS1, SOCS3 utilizes a dual-binding mechanism to simultaneously engage both gp130 (IL-6 receptor subunit) and JAK, thereby reducing JAK activity via obstruction of the substrate binding site[19].

### Other SOCS proteins

SOCS4 and SOCS5 have similar functions in regulating EGFR signaling. It has been shown that SOCS4 expression can be induced by EGF, followed by the binding of SOCS4 to phosphotyrosine residues on activated EGFR, thereby reducing STAT3 signaling [20]. As with SOCS4, SOCS5 in vitro, where promotes EGFR degradation through SOCS box recruitment of the E3 ligase complex[20]. In addition to negatively regulating the EGFR signaling pathway, SOCS5 reduced JAK phosphorylation, via direct interaction between JAK1- and JAK2-conserved JH1 domains[21]. Additionally, SOCS5 regulated the EGFR-PI3K signaling pathway by direct binding with EGFR, leading to the suppression of pro-inflammatory responses and enhanced antiviral immunity.

The suppression of cytokine signaling pathways by SOCS molecules occurs through three approaches. (Ⅰ) IFN-β can trigger the JAK-STAT1 pathway. SOCS1 negatively regulates IFN-β signaling and inhibits the generation of CD40, iNOS, and interferon-stimulated genes (ISGs). IL-6 activates STAT1 and STAT3 to promote inflammatory processes, while activated STATs induce SOCS3 expression, thereby terminating the signaling cascade. (Ⅱ) SOCS2 can compete with SHP2, maintaining sustained EGFR activation, and thereby inducing neurite growth. (Ⅲ) The stimulation of TLR4 by LPS initiates the JAK2-STAT5 pathway, which can be suppressed by SOCS1. TRAF6 activates the TAK1-MAPK pathway via MyD88/MAL, whereas SOCS1 negatively regulates this signaling by binding phosphorylated MAL and mediating its ubiquitination degradation. SOCS1 also modulates mitogen-activated protein kinase (MAPK) signaling through interaction with apoptosis signal-regulating kinase 1 (ASK1). (Ⅳ) The role of SOCS proteins in the crosstalk of IL-4/STAT6 and IL-12/STAT4 signal pathways.

## The regulation of SOCS family members

As key regulators of cytokine signaling, the SOCS family plays a central role in inflammatory response, immune homeostasis, and cellular differentiation through a multilevel dynamic regulatory mechanism. Studies have shown that the functions of SOCS proteins are not only precisely regulated by gene transcription, translation, and post-translational modifications but also by a complex network of interactions among their family members[5,14].

### Transcription

SOCS3 expression is regulated by the IL-6 signaling pathway and mRNA ubiquitination sites. In the IL-6 signaling pathway, the activation of STAT3 leads to the transcription of SOCS3, which in turn inhibits further STAT3 activation by binding to the YxxV motif (Y759) within the intracellular domain of gp130[22]. This process recruits the E3 ubiquitin ligase complex, thereby facilitating the ubiquitination and subsequent degradation of JAK kinases[22]. This mechanism establishes a negative feedback loop that serves to regulate the duration of inflammatory signaling. Furthermore, SOCS3 has alternate mRNA initiation, leading to the absence of the exon that encodes the ubiquitination site, thereby enhancing the degradation of SOCS3[23].

### Translation

SOCS mRNA expression levels are regulated by eukaryotic initiation factor 4E binding protein and M^6^A modifications. Expression of SOCS1 exhibits significant repression at the translation initiation stage by eukaryotic initiation factor 4E-binding proteins[24]. Moreover, in T cells, IL-7 signaling accelerates the degradation of SOCS1 and SOCS3 mRNA by inducing m⁶A modification of SOCS mRNA, which reduces SOCS protein levels, deregulates the IL-7/STAT5 pathway, and ultimately promotes homeostatic proliferation and differentiation of T cells[25].

### Post-translation

SOCS members can be regulated post-translationally[26]. One example is the interaction between SOCS1 and Pim kinases, which phosphorylates and stabilizes the SOCS1 protein, prolonging its half-life and its ability to block JAK-STAT activation[26]. Notably, the coexpression of tripartite motif-containing 8 (TRIM8) with SOCS1 impairs the stability of SOCS1, which is associated with reducing JAK-STAT activation[27]. Another mechanism of post-translational regulation involves changes in subcellular localization[28,29]. Recent studies have revealed that CISH, SOCS1, SOCS2, SOCS3, SOCS6, and SOCS7 molecules are capable of being translocated to the nucleus[[28], [29], [30], [31], [32]], where they may enhance the suppression of cytokine signaling[30]. Besides nuclear translocation, SOCS1 binds to the microtubule adaptor protein MAP1S and the microtubule organizing complex, which may further potentiate the inhibition of cytokine signaling[31,32].

### Crosstalk of SOCS proteins

Emerging evidence suggests that SOCS proteins may disrupt the inhibitory function of other family members in a cross-regulatory mechanism[[33], [34], [35]]. SOCS2 seems to promote the degradation of SOCS1, SOCS3, and CISH[33]. While SOCS1, SOCS3, and CISH are usually induced rapidly and transiently upon receptor activation, SOCS2 expression often occurs later and is maintained for a prolonged period following cytokine stimulation[34]. Following receptor stimulation, SOCS2 accumulation may help remove excess SOCS protein, preserving cell sensitivity to stimulation[35]. Interestingly, SOCS2, SOCS6, and SOCS7 exhibit the ability to bind to one another, indicating a potential mechanism for self-elimination[33]. A comprehensive analysis of the interaction patterns of SOCS proteins with cytokine receptors and their self-interactions is essential for a deeper understanding of their specific inhibitory properties.

## Roles of the SOCS family in immune cells

Research indicates that impaired expression of SOCS proteins significantly impacts the maturation and functionality of innate and adaptive immune cells, highlighting the crucial regulatory roles of SOCS proteins in various immune cell types[36,37] (Table 2).

**Table 2:** The Role of the SOCS family in immune cells.

**Cell Type**	**SOCS**	**Function**	**Ref**
DCs	SOCS1	DCs deficient in SOCS1 are more sensitive to TLR ligand stimulation.	[36]
	SOCS2	Limits DC-based T cell priming and antigen-specific adaptive immunity.	[42]
	SOCS3	suppresses DC differentiation and DC-mediated anti-tumor T cell responses	[45]
	CISH	CISH knockdown impaires DC-based tumor immunotherapy.	[46]
Macro-phages	SOCS1	Increases the polarization of M2 macrophages.	[19,54,56]
	SOCS2	Suppresses inflammatory factors, including TNF-α, IL-1β, IL-6. (RAW 264.7 cell)	[58]
	SOCS3	Suppresses LPS-mediated CD40 expression	[180]
	CISH	CISH-deficient macrophages show defective function.	[48]
NK cells	SOCS2	Decreases NK cell numbers.	[65]
	SOCS3	Impaires IL-15 receptor signaling.	[66]
	CISH	Inhibits NK cell survival, proliferation, and cytotoxicity.	[32]
MDSCs	SOCS3	Inhibits the development and function of MDSCs	[70,72]
CD4^+^ T cells	SOCS1	Inhibits the activation and differentiation of CD4^+^ T cells.	[75,80,[91], [92], [93]]
	SOCS2	Upregulated in Treg cells and regulates IL-2 signaling and Treg cell development.	[89]
	SOCS3	Promotes Th2 differentiation, inhibits Th17 differentiation and Treg function.	[9,81,85,86,90]
	CISH	Promotes TCR- mediated proliferation and cytokine production.	[94]
CD8^+^ T cells	SOCS1	Blocks IL-7 signaling in naïve CD8^+^ T cells.	[180]
	SOCS2	Inhibits activation of CD8^+^ T lymphocytes.	[42]
	SOCS3	Suppresses CD8^+^ T cell proliferation.	[101,104]
	CISH	Deleting CISH in CD8^+^ T cells enhances their proliferation and functionality.	[20]
B cells	SOCS1	Involved in IL-15 modulates the B1a cell response	[109]
	SOCS3	Enhances progenitor B cell growth and differentiation in bone marrow. Promotes the exit of immature B cells and the circulation of mature B cells.	[106]

### Innate immune cells

Innate immune cells, which comprise dendritic cells (DCs), macrophages, natural killer (NK) cells, and myeloid-derived suppressive cells (MDSCs) that detect antigens like LPS and CpGs, activate Toll-like receptors (TLRs), which are the first line of defense against pathogens[38,39].

#### Dendritic cells

DCs function as cells that present antigens that facilitate the connection between innate and adaptive immunity, exerting a vital part in the production and control of immune responses[40]. Sharon et al. reported that SOCS1, SOCS3, and CISH are constitutively elevated at relatively minimal levels in plasmacytoid DCs and that these levels are dramatically upregulated as the cells differentiate into myeloid DCs, suggesting that SOCS protein expression may be critically involved in DC maturation[41].

SOCS family members, including SOCS1, SOCS2, and SOCS3, regulate DC function by restricting antigen presentation capacity, suppressing T cell proliferation, and ultimately reducing the production of proinflammatory cytokines[37,[42], [43], [44]]. Compared to DCs expressing SOCS1, DCs lacking SOCS1 exhibit greater antigen presentation ability, T cell proliferation, and sensitivity to TLR ligand stimulation ex vivo, leading to increased secretion of proinflammatory cytokines[37,42]. Chen et al. found that DC antigen presentation and DC-mediated antitumor immunity were strengthened when SOCS1 was silenced[42]. When SOCS1 is selectively deleted from DCs in vivo, fatal autoimmune CD8^+^ T cell responses are primed, ultimately disrupting self-tolerance and driving effective anti-tumor actions[45]. Additionally, SOCS2 deficiency enhances anti-tumor immunity by enhancing DC-mediated T-cell priming and adaptive immunity[43]. Similarly, SOCS3 in DC precursors suppresses DC differentiation and DC-caused antitumor T cell responses[44]. Moreover, the combination of SOCS3 with pyruvate kinase type M2 (M2-PK) reduces M2-PK activity, a crucial enzyme in the glycolytic metabolic process, consequently impairs the antigen-presenting ability of DCs[46]. Inconsistent with the SOCS proteins described above, CISH knockdown increased DC production via cell-cycle activation and cell apoptosis reduction but impaired DC-based tumor immunotherapy by decreased Cytotoxic T lymphocyte (CTLs) generation[47].

Collectively, expressing different SOCS proteins appears to have different effects on DCs. SOCS1, SOCS2, and SOCS3 can impair DC function, whereas CISH expression in DCs promotes DC-mediated CD8^+^ T-cell immunity.

#### Macrophages

Macrophages are diverse cells crucial for tissue homeostasis and inflammation, capable of destroying pathogens and tumor cells and later promoting tissue repair and healing[48]. Although the expression of SOCS protein is initially low in macrophages, SOCS1, SOCS3, and CISH can be rapidly induced by IFN-γ, IL-6, and GM-CSF and regulated M1 and M2 macrophage polarization[16,[49], [50], [51], [52], [53]].

**Fig. 3:**
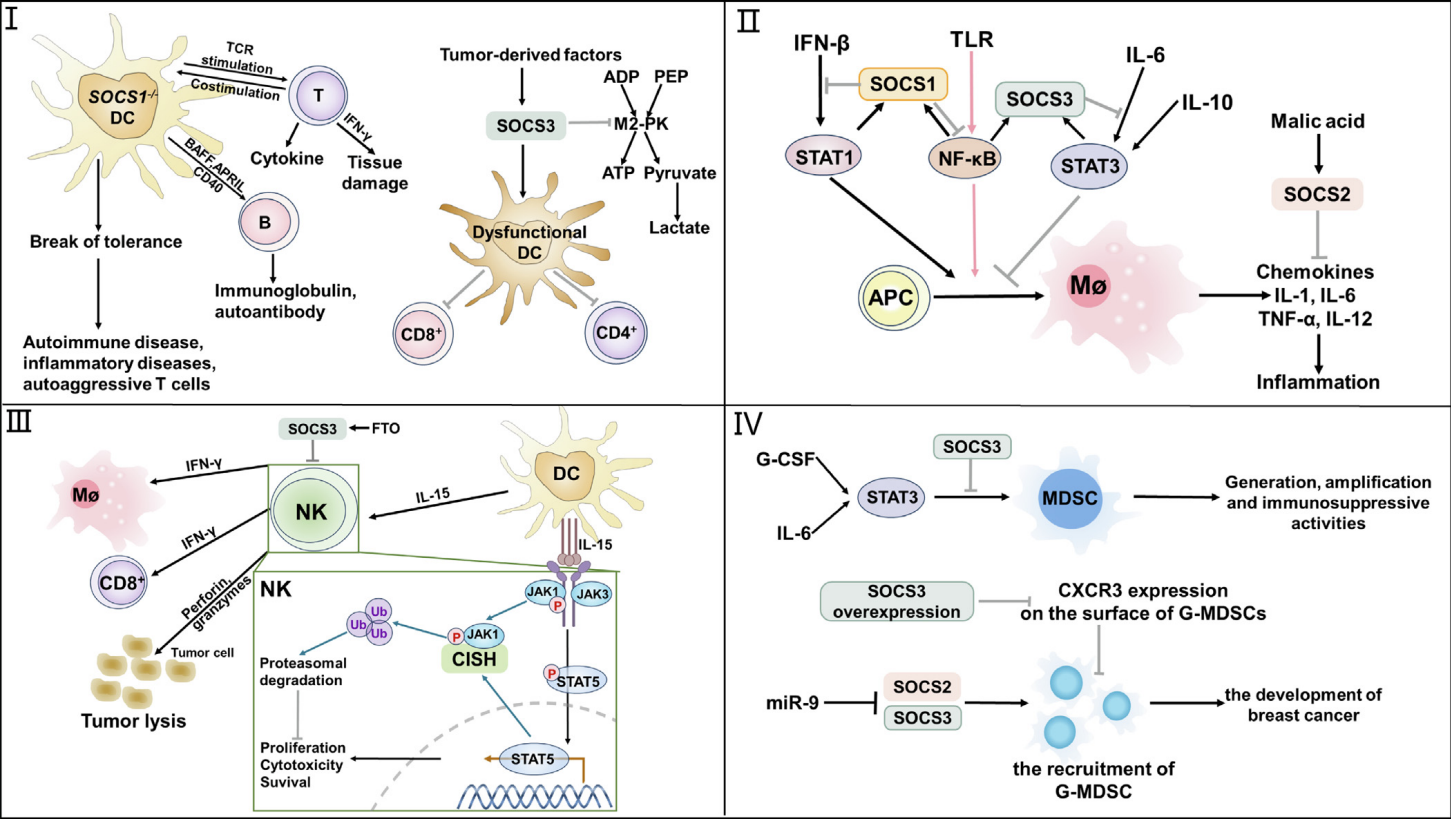
The Role of the SOCS family in innate immune cells.

SOCS1 is critical in macrophage polarization by regulating multiple mechanisms, significantly affecting immune responses[54,55]. The expression of the M1 gene, such as TNF-α and IL-12p40, is elevated in macrophages with SOCS1 deficiency in response to LPS stimulation[54]. MAL is a part of the Toll/IL-1 receptor (TIR) domain-containing adapter proteins (TIRAP) family involved in TLR signaling and necessary for the generation of M1 cytokine after LPS stimulation[56]. Research indicates that following TLR2 and TLR4 activation, SOCS1 interacts with phosphorylated MAL, leading to the polyubiquitination and degradation of MAL, while elimination of SOCS1 increases MAL-dependent p65 phosphorylation and nuclear factor kappa-B (NF-κB) activation, which intensifies responses of inflammation[16,57]. Besides, by directly binding to NF-κB p65, SOCS1 causes its proteolysis process and inhibits NF-κB activation[55]. Thus, SOCS1 deficiency is essential for initiating the transcription regulation of pro-inflammatory genes, which relies on the TLR-NF-κB pathway (Fig. 2). The blockade of the JAK-STAT pathway is another significant method through which SOCS1 suppresses macrophage activation. For instance, IFN-β promotes JAK1/STAT1 signaling to produce the costimulatory molecule CD40, essential for effective antigen presentation to T cells, resulting in T cell activation[58]. Meanwhile, STAT1 promotes the development of the SOCS1, which operates as the adverse regulation feedback mechanism to suppress IFN-β signaling, hence reducing CD40 expression[58] (Fig. 3). In summary, SOCS1 coordinates the modulation of different cellular pathways to regulate macrophage polarization and function, maintaining immune system homeostasis.

SOCS2 in macrophages is crucial for dampening the pro-inflammatory response by suppressing proinflammatory mediators, such as TNF-α, IL-1β, IL-6, and IL-8, consequently limiting the activation of NF-κB[59]. Additionally, SOCS2 can reduce the levels of IL-17 and IL-18 by preventing the activation of NOD-like receptor thermal protein domain associated protein 3 (NLRP3)[59]. Studies have shown that L-Malic acid (MA), an intermediate in the tricarboxylic acid cycle, can increase SOCS2 expression, thereby inducing M2 macrophage polarization, leading to the recovery of injured tissues[60].

SOCS3 serves as a crucial modulator of gp130 in macrophages, and SOCS3 converts IL-6-induced pro-inflammatory response to IL-10-like anti-inflammatory response[50]. A rational explanation is that IL-6 and IL-10 increase SOCS3 expression when LPS is present. However, SOCS3 binds to the IL-6R subunit gp130 instead of IL-10R, leading to IL-6 signaling being selectively blocked[50,61] (Fig. 3).

CISH was elevated in alveolar macrophages (AMs) following GM-CSF stimulation[49]. CISH-deficient bone marrow (BM) derived macrophages exhibited increased lipids, with increased GATA binding protein 2 (GATA2) expression, which plays a key role in maintaining lung homeostasis[49]. These results indicate that CISH constrains GATA2 expression to preserve AM identification and functionality.

In conclusion, targeting SOCS proteins in macrophages is viable for treating cytokine-induced inflammatory disorders.

#### NK cells

NK cells primarily function in innate immunity and significantly influence adaptive immune responses by exhibiting immediate cytotoxicity and rapidly producing cytokines and chemokines to sustain inflammation[62]. IL-15, an element of the IL-2 family, is essential for NK cell development and cytotoxic function[63]. CISH can interact with JAK1 for proteasomal degradation and abrogating IL-15 signaling. SOCS2, which is associated with CISH, can be induced by Growth Hormone (GH) and IL-15, activating STAT5 in mouse and human NK cells[64]^-^[65]. Removal of CISH leads to a heightened level of IL-2Rβ and augmented expression and activity of JAK1 and JAK3, triggering NK cell survival, proliferation, and cytotoxicity[36], while SOCS2^-/-^ mice show elevated NK cell populations with no significant changes in cytotoxicity or IFN-γ production[66]. Thus, CISH and SOCS2 regulate NK cell function by modulating IL-15-induced signaling.

In murine NK cells, SOCS3 is upregulated by IL-15 signaling and acts as a target gene of the inhibitor of DNA binding 2 (ID2), which is crucial for NK cell development[36,67]. When ID2 was lost in mature NK cells, SOCS3 genes were upregulated, which led to a decrease in NK cell survival by causing IL-15 receptor hyporesponsiveness[67]. Through enhancing the expression of SOCS3, the m6^A^ demethylase fat mass and obesity-associated protein (FTO) inhibits JAK/STAT activation by IL-15[68]. SOCS1 suppressed by miR-155 is crucial for the proliferation of virus-targeting natural killer cells in the face of MCMV infection, with the underlying mechanisms yet to be fully elucidated[69].

Thus, the available investigations have shown that the SOCS proteins are implicated in NK cell development, survival, proliferation, and cytotoxicity. SOCS proteins could serve as immune checkpoints for NK cells, providing the foundation for novel immunotherapeutic strategies in NK cell-based therapies.

#### Myeloid-derived suppressor cells

MDSCs are immature myeloid cells that can be recruited into the tumor microenvironment during cancer development and reduce the function of anti-tumor T lymphocytes[70]. SOCS3 suppresses the growth and function of MDSCs in prostate cancer, while the absence of SOCS3 in myeloid cells exhibits heightened STAT3 activation and facilitates progenitor cell differentiation into MDSC phenotype and the generation of immunosuppressive microenvironment[71]. In accordance with the findings mentioned above, another study revealed that SOCS3 was markedly diminished in MDSCs derived from primary breast cancer and in vitro produced MDSCs, which was substantially associated with prolonged stimulation of the JAK-STAT pathway and enhanced immunosuppression of T cells in MDSCs[72]. The failure of SOCS3 activated by IL-6 and prolonged stimulation of the JAK/STAT signaling pathway led to the proliferation of MDSCs and augmented their immune suppressive impact on the immunity of T cells in vitro[72]. Consequently, SOCS3 deficiencies may be the primary factor underlying IL-6-induced prolonged stimulation of the JAK-STAT signaling, leading to increased differentiation and immunosuppressive function of MDSCs. It has been shown that SOCS2 and SOCS3 are identified as target genes of miR-9[73]. SOCS3 overexpression inhibited the expression of C-X-C chemokine receptor type 3 (CXCR3), dependent on miR-9, on the surface of G-MDSCs, thereby inhibiting the development of breast cancer[73]. To sum up, these findings emphasized the significance of SOCS3 in limiting MDSC-mediated suppressive activity in tumors.

SOCS molecules exert crucial regulatory roles in different types of immune cells.

(Ⅰ) SOCS1-deficient DCs exhibit hyperactivation, leading to tissue damage, autoimmune diseases, and inflammatory diseases. Tumor-derived factors boost SOCS3, combining with M2-PK to reduce ATP generation, resulting in DC dysfunctional. (Ⅱ) In macrophages, SOCS1 suppresses STAT1 and NF-κB, thus preventing inflammatory factor production. IL-6 and IL-10 highly induce STAT3 generation, but IL-6 signaling is specifically blocked by SOCS3. Malate increases SOCS2 expression, reducing M1 macrophage gene production. (Ⅲ) CISH interacts with JAK1 for proteasomal degradation, thereby restraining IL-15-involved development, survival, proliferation, and cytotoxicity functions of NK cells. FTO inhibits JAK/STAT activation by IL-15 through enhancing SOCS3 expression. (Ⅳ) SOCS2 and SOCS3 modulate MDSC differentiation and function involved in tumor progression.

### Adaptive immune cells

#### T cells

**Fig. 4:**
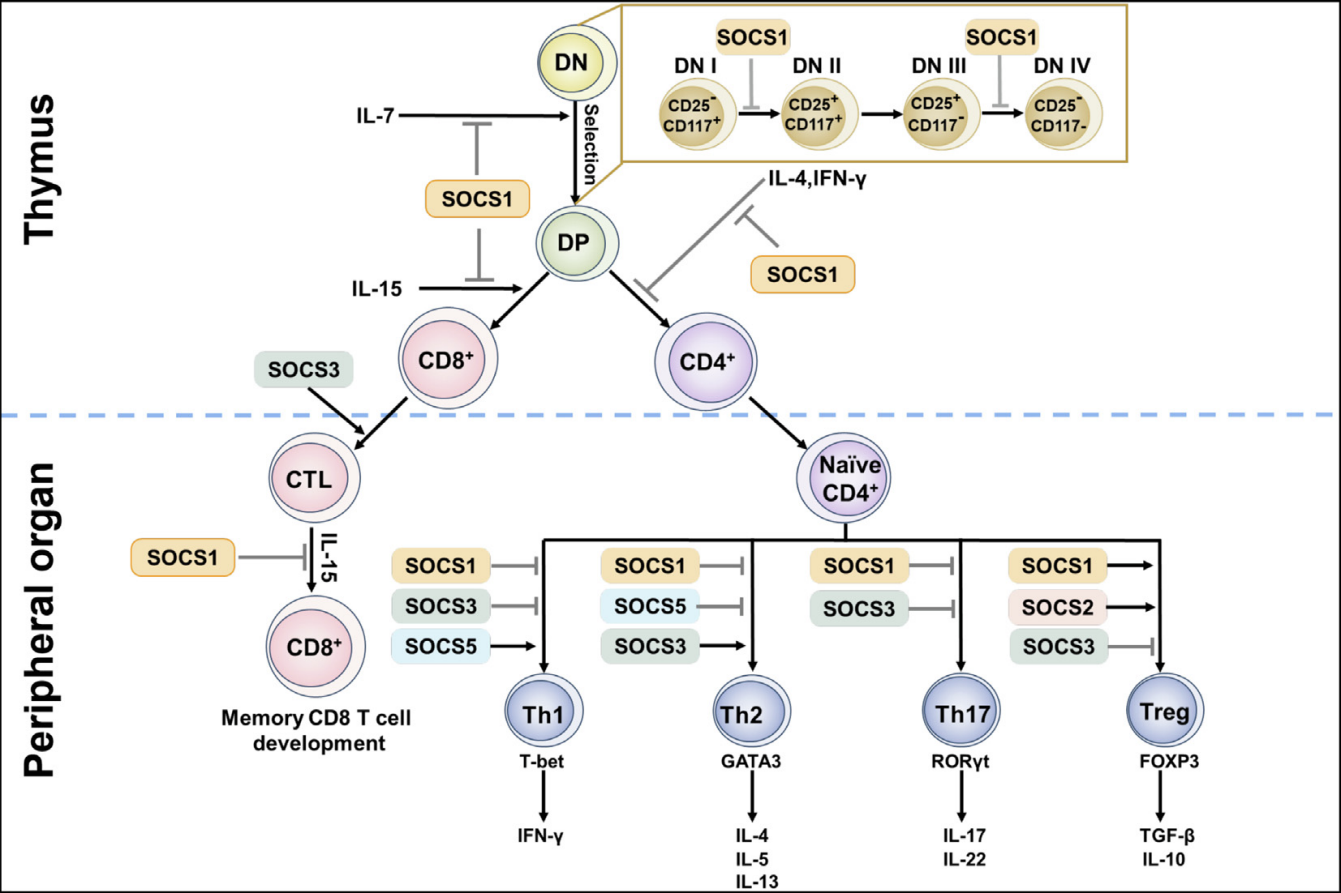
The Role of the SOCS family in adaptive immune cells.

CD4^-^CD8^-^ (double-negative, DN) can be subdivided into subset CD25^-^CD117^+^ (DNI), CD25^+^CD117^+^ (DNII), CD25^+^CD117^-^ (DNIII), and CD25^-^CD117^-^ (DNIV), which is based on the CD25 (IL-2Rα) and CD117 (c-Kit) expression[74,75]. SOCS1 is expressed across all thymic developmental stages[[75], [76], [77]]. SOCS1 regulates T cell development by inhibiting cell expansion at the DNII stage through modulation of c-Kit and γc signals and suppressing cell development during the transition from the DNIII to the CD4^+^CD8^+^ (double positive, DP) stage[75]. Additionally, The expression of SOCS1 is temporarily reduced during pre-TCR signaling, facilitating β-selection in the conversion of DNIII cells to DP cells[75] (Fig. 4).

##### CD4^+^ T cells

Recent evidence has shown that the SOCS family significantly influences CD4^+^ T cell development[14,78]. Knockdown of SOCS1 in CD4^+^ T cells increases Th1 cell generation[79], whereas SOCS1 overexpression inhibits the generation of Th1 cells[80]. Consistently, upon stimulation with anti-CD3, SOCS1^-/-^ CD4^+^ T cells consistently generated greater quantities of IFN-γ and IL-4 than wild-type controls, indicating that SOCS1 negatively modulates Th1 and Th2 cell development in the presence of IL-12 and IL-4, respectively[81]. IL-12 activates STAT4 to promote Th1 cell differentiation. SOCS3 is highly expressed in Th2 cells and connects to the STAT4 docking site in IL-12Rβ2, thus inhibiting IL-12 signaling, leading to suppressing Th1 cell differentiation and promoting a Th2-skewed phenotype[82,83]. Furthermore, conditional deletion of SOCS3 in T cells reduced Th2 cell responses due to activation of STAT3 to elevated generation of TGF-β and IL-10[84]. IL-4 signaling through STAT6 induces GATA binding protein 3 (GATA3) expression, increasing Th2 cell development[85]. The cytoplasmic region of IL-4Rα has been found to interact with SOCS5, preventing the recruitment of STAT6 and reducing GATA3 expression, thereby inhibiting Th2 differentiation[85]. Thus, SOCS proteins are essential for maintaining the balance of Th1/Th2 responses (Fig. 2). STAT3 binds to promoters of IL-17A and IL-17F, enhancing Th17 generation[86]. SOCS3 inhibits Th17 differentiation by blocking STAT3 signaling[87], and the deletion of SOCS3 in T cells increases IL-17 production[88]. Similarly, SOCS1 suppresses IL-17 production, thereby inhibiting Th17 cell differentiation[89]. Treg cells, distinguished by Forkhead transcription factor (FOXP3) expression, are essential for immunological tolerance, safeguarding the host from excessive immune reactions[90]. SOCS1 defends Treg cells from the detrimental impact of pro-inflammatory cytokines by maintaining Foxp3 expression[89]. It has been shown that SOCS2 was upregulated in Treg cells, enhancing IL-2 signaling and Treg cell development[91]. Unstimulated Treg cells exhibit minimal SOCS3 expression, and SOCS3 overexpression hampers Treg cell development and functionality[92]. Thus, different SOCS molecules exhibit distinct functions, leading to diverse regulatory roles in CD4^+^ T cell development (Fig. 4).

Apart from its function in cytokine-induced T-cell differentiation, the SH2 domain of SOCS1 has been demonstrated to bind with phosphorylated immunoreceptor tyrosine-based activation motifs (ITAMs) of CD3z and Syk[93]. The production of SOCS1in response to IL-2 activation and its association with elements of the TCR signaling cascades, including Grb2, Vav1, p85, and Itk, indicate that SOCS1 is probably involved in regulating T cell receptor (TCR) signaling[76,94,95]. Additionally, CISH expression is specifically triggered in T cells following activation of the TCR[96]. Moreover, increased CISH significantly enhances TCR-mediated growth and cytokine generation in vitro and extends the longevity of CD4^+^ T cells upon TCR activation[96]. Taken together, SOCS proteins, like SOCS1 and CISH, modulate TCR signaling, potentially playing a crucial role in the regulation of immune response and balance.

##### CD8^+^ T cells

SOCS1 prevents the uncontrolled proliferation of CD8^+^CD44 ^high^ memory T cells and promotes the survival of CD8^+^CD44^low^ naive T cells, thereby modulating CD8 cell development[[97], [98], [99]]. IL-7 and IL-15 signaling ensure the maintenance of the CD8^+^ naive state during the transition from DP to CD8 single-positive (CD8SP) cells[100,101]. Research indicates that CD8 cells of SOCS1-deficient mice exhibit hypersensitivity to IL-7 and elevated differentiation into CD8SP cells attributable to elevated STAT5 phosphorylation, characterized by a memory phenotype with elevated CD44 expression and diminished CD25 and CD69 expression[99,100]. Additionally, IL-15 induces the activation and proliferation of naive CD8^+^ T cells, and SOCS1 prevents the hyperactivation of naive CD8^+^ T cells by inhibiting IL-15 signaling[102]. The data mentioned above elucidate that SOCS1 plays a crucial role in regulating CD8^+^ T cells by controlling development and maintaining naive phenotype[5,78]. Lung can serve as an athymus tissue for the BM cells to develop into CD8^+^ T lymphocytes[103]. IL-6, LPS, and tumor-derived factors significantly enhance SOCS3 expression in BM cells, enabling SOCS3 to facilitate the development of BM cells into CD8^+^ T cells by increasing Notch1 expression, which helps CD8^+^ T cells differentiate into effector cells in some pathologic conditions,such as tumor and inflammation,[103].

SOCS proteins, including SOCS1, SOCS2, SOCS3, and CISH, suppress CD8^+^ T activation, thereby promoting tumor growth[104,105]. Evidence indicates that SOCS1 silencing augmented tumor destruction, with elevated miR-155, which is crucial for anti-tumor immunotherapy[105]. Moreover, SOCS2 inhibited activation of CD8^+^ T lymphocytes, and mice deficient in SOCS2 exhibited increased CD8 infiltration and enhanced antitumor development[43]. Additionally, T cells lacking SOCS3 showed enhanced hyperactivation caused by augmented IL-27, which promotes CD8^+^ T cell activation[106]. Similarly, CISH binds to the TCR mediator PLC-γ1, facilitating its proteasomal degradation following TCR activation, inhibiting activation of TCR signaling and CD8^+^ T cell, thereby blocking anti-tumor activity[17]. These findings suggest that targeting SOCS molecules provides new ideas and possibilities for cancer treatment and applications in tumor immunotherapy.

SOCS1 is involved in early T cell development in the thymus and peripheral organs. Different SOCS proteins play distinct roles in differentiating naive CD4^+^ T cells into various T cell subsets.

#### B cells

Pathogenesis can be facilitated by B cells or converted plasma cells in many ways, such as matrix metalloproteinase-3 synthesis, cytokine generation, and T cell stimulation[107]. SOCS3 expression is modest in progenitor B cells but rises during the immature B cell stage, attaining its peak in splenic B cells[108]. During B lymphopoiesis, reduced SOCS3 expression facilitates the retention of progenitor B cells within BM niches for extended growth and differentiation, while elevated SOCS3 expression encourages the migration of immature B cells and the movement of mature B cells by attenuating C-X-C motif chemokine ligand 12 (CXCL12)-activated focal adhesion kinase (FAK) stimulation and adhesive responses in B lineage cells within the BM[108]. Additionally, it has been observed that the maintenance of SOCS3-deficient GC B cells was impaired, suggesting that SOCS3 has a stage-specific role in differentiating B lymphopoiesis[109].

It has been shown that SOCS1 performs a distinctive part in controlling IL-15-mediated signaling in B1a cells, the main proportion of B cells in the innate immune system that produce natural antibodies against external invaders[110]. IL-15 modulates the B1a cell response by curbing the initial amplification of IL-10 through SOCS1 upregulation because IL-10 must remain elevated to allow B1a cells to survive long-term in vitro culture via the generation of the hallmark cell marker CD5[110,111]. Interestingly, B1a cells not only decreased their expression of the anti-inflammatory cytokine with the increase in SOCS1 but also tended toward a pro-inflammatory response[112]. Thus, the available investigations have shown that SOCS1 is crucial for B cell survival and modulating the mucosal immune response of B1a cells. Mice infected with the Abelson murine leukemia virus (A-MuLV) exhibit v-Abl oncogene expression that induces sustained JAK-STAT signaling, leading to B cell lymphoma due to the failure of SOCS1 to inhibit JAK-STAT activity[113,114]. A mechanism for this is that v-Abl signaling activated SOCS1 phosphorylation and suppressed SOCS1-dependent degradation of JAKs[114]. Moreover, the formation of the E3 ubiquitin ligase complex between SOCS1 and Elongin C is impaired, resulting in SOCS1 dysfunction[114]. The aforementioned data elucidate a unique way v-Abl may avoid SOCS1 suppression in B cell lymphoma.

## SOCS family in inflammation and autoimmune pathogenesis

**Fig. 5:**
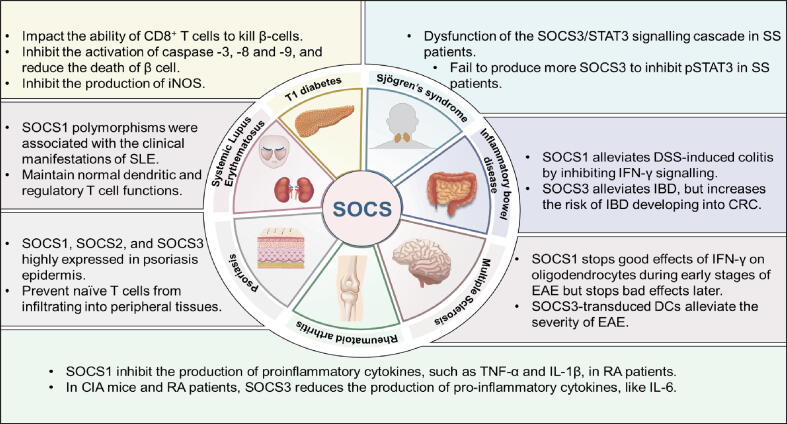
The Role of the SOCS family in autoimmune diseases.

According to recent studies, defective expression of the SOCS molecules could impact the development and function of innate and adaptive immune cells, revealing a significant role of the SOCS family in autoimmune disorders[115,116]. Moreover, SOCS1 peptide mimetic and SOCS3 overexpression by adenoviral vectors have effectively alleviated autoimmune diseases[117,118]. This section addresses recent advancements in the immune-regulatory effect of SOCS in developing autoimmune diseases (Fig. 5).

### Rheumatoid arthritis

Rheumatoid arthritis (RA) is a chronic autoimmune inflammatory joint disorder, mainly distinguished by joint tissue damage[119]. Studies have shown that SOCS1 and SOCS3 levels are elevated in RA patients[120]. Moreover, compared to healthy controls, SOCS1 is predominantly upregulated among T cells, while SOCS3 is mostly increased in monocytes in RA patients[120]. Previous studies have shown that SOCS1 inhibits pro-inflammatory cytokine generation, such as TNF-α and IL-1β, in RA patients[121]. Similarly, SOCS3 decreased the generation of pro-inflammatory cytokines, like IL-6, in RA patients and collagen-induced arthritis (CIA) mice[118]. Furthermore, treatment of adenovirus containing SOCS3 led to inhibition of bone destruction and alleviation of inflammation in CIA mice[118].

Research indicates that a deficit in SOCS3 correlates with joint disease. For instance, compared with wide-type (WT) mice, mice with SOCS3 deficiency in hematopoietic and endothelial cells (SOCS3^-/vav^) showed exacerbated IL-1-induced acute inflammatory arthritis, distinguished by a significant neutrophilic synovial infiltration and raised bone deterioration[122]. gp130^F759^ mice with tyrosine residue Y759 of gp130 mutated to phenylalanine (F759) spontaneously develop an RA-like phenotype, which is attributed to the inability of SOCS3 to negatively regulate gp130-stimulated STAT3 activation after the Y759 mutation[123]. Another mutant mice deleted all STAT binding sites named gp130^△STAT^, which possessed joint conditions that feature persistent synovitis, cartilaginous metaplasia, and deterioration of the articular cartilage[124]. The constant stimulation of the SHP2/Ras/Erk pathway through the receptor subunit gp130, resulting from the mutation of the STAT binding site, leads to enhanced responsiveness and proliferation of synovial fibroblasts from mutant animals to IL-6[124]. This phenomenon is accompanied by the complete absence of SOCS1 expression and reduced SOCS3 expression[124]. Furthermore, elevated levels of SOCS1 protein can suppress IL-6 signaling in synovial cells of gp130^△STAT^ mice to counteract the deficient SOCS1 and SOCS3 expression[124]. Collectively, these results emphasize the critical function of SOCS proteins in joint pathology and highlight that targeting SOCS1 and SOCS3 may constitute a practical method for treating RA.

### Systemic lupus erythematosus

Systemic lupus erythematosus (SLE) is an autoimmune disorder defined by multiple tissue and organ damage due to the deposition of immune complexes[125]. The level of SOCS1 mRNA was markedly elevated in SLE patients, with even higher levels observed in those with active SLE compared to inactive cases[126]. Moreover, SLE patients with central nerve system (CNS) involvement demonstrated a greater prevalence of SOCS1-1478del/del than those without CNS involvement[126]. However, in dominant gene-driven SLE patients, the prevalence of SOCS1-1478del/del is reduced in patients with thrombocytopenia[126]. These findings suggested that the SOCS1 polymorphism is associated with SLE clinical symptoms[126]. Studies have shown that SLE patients displayed increased SOCS3 expression compared to healthy controls[127]. Furthermore, conditional knockout of JunB in the skin to generate JunB^△ep^ mice results in a spontaneous SLE phenotype development, which is dependent on increased epidermal IL-6 secretion, accompanied by elevated SOCS3 protein expression, suggesting that elevated SOCS3 levels observed in SLE are not sufficient to inhibit the sustained IL-6 stimulation[127].

Studies indicate that SOCS1 is crucial for maintaining normal dendritic and Treg cell functions. Mice with SOCS1 deficiency in all cells except T cells and B cells (SOCS1^-/-^-Tg mice) spontaneously show increased serum anti-DNA autoantibodies and glomerulonephritis with glomerular IgG deposition[37]. These phenotypes resemble those of murine models for SLE. This can be attributed to the increased sensitivity of SOCS1-deficient DCs to TLR ligand stimulation, which consequently results in augmented antigen-presenting capacity and T-cell proliferation, ultimately leading to the development of SLE[37]. Moreover, a study has shown that mice with a single SOCS1 allele defect (SOCS1^+/-^ mice) spontaneously developed an SLE-like phenotype[128]. This is attributable to a reduction in SOCS1, which results in insufficient down-regulation of IL-2 signaling, thereby impairing Treg suppressive function[128]. These findings suggest that SOCS1 is crucial in regulating immune responses and maintaining homeostasis in SLE.

Therapeutic agents based on SOCS1 have shown promise in reducing the severity of SLE[[129], [130], [131]]. A study has shown that hCDR1, a peptide derived from a pattern of the initial complementarity determining region (CDR1) of an autoantibody, can induce SOCS1[129]. Administration of hCDR1 to lupus mice (NZB × NZW) improved the condition, evidenced by diminished autoantibody synthesis, lower proteinuria levels, and reduced glomerular immune complex deposits[130]. Similarly, Sharma et al.[131] found that treating SOCS1 kinase inhibitory region (SOCS1-KIR) peptide mimic alleviated disease severity in MRL/lpr mice, which spontaneously generated lupus-like disease[131]. In sum, SOCS1-KIR and SOCS1 mimetic peptides may represent new approaches for treating SLE.

### Multiple sclerosis

Multiple sclerosis (MS) is an autoimmune neurodegenerative disease manifested by activated macrophages and microglia in the CNS that express abnormal cytokines levels, like IL-1β, IL-6, and IL-23, and chemokines CCL3, CCL4, and CCL5, which lead to neuronal damage and demyelination[132]. SOCS1 has been observed to play both helpful and harmful roles in MS progression. The specific impact of SOCS1 appears to be contingent upon the prevailing inflammatory microenvironment or disease context. Research has shown that in the initial phases of experimental allergic encephalomyelitis (EAE), a model for MS, SOCS1 inhibits the beneficial effects of IFN-γ in oligodendrocytes while preventing its detrimental actions later in the disease process[133,134]. This is because, before the occurrence of EAE, IFN-γ can potentially improve the disease and prevent the initiation of demyelination, axonal damage, and oligodendrocyte loss[135]. However, IFN-γ has also been observed to upregulate CD40 expression on the surface of macrophages and microglia at a later stage, which can be deleterious[136].

Similarly, SOCS2 is detrimental at the onset of EAE but beneficial later in the disease stage[137,138]. Studies have shown that, compared to WT mice, SOCS2^-/-^mice showed strong resistance to the development of EAE during early EAE development[137]. However, after the peak of the disease, SOCS2^-/-^ mice failed to recover from CNS damage, and EAE disease scores continued to increase[137]. The mechanisms need to be further explored. Furthermore, IFN-β treatment in WT-EAE mice can limit NF-κB activation by upregulating SOCS2 expression, ultimately reducing EAE disease scores[138]. Thus, understanding the role of SOCS1 and SOCS2 in different disease stages could facilitate the development of precision treatments for MS.

Relapsing-remitting multiple sclerosis (RR-MS) patients exhibit monocytes, CD4^+^ T cells, and CD8^+^ T cells that produce higher p-STAT3 and fewer SOCS3 throughout recurrence than during recovery, indicating a connection between reduced SOCS3 expression, more STAT3 activation, and MS relapse[139]. A recent study found that simvastatin, which is used to control cholesterol levels, also has immunomodulatory effects by increasing the expression of SOCS3 and SOCS7 and inhibiting the generation of IL-6 and IL-23 in monocytes from patients with relapsing MS[140,141]. SOCS3 exerts a protective role against EAE. IFN-β induced SOCS3 expression in astrocytes, while SOCS3 deficiency in astrocytes leads to increased expression of the chemokines CCL2, CCL3, CCL4, CCL5, and CXCL10, thereby recruiting macrophages and CD4^+^T cells to inflammation region[142]. Moreover, injection of SOCS3-transduced DCs significantly suppressed the generation of IFN-γ and IL-17, consequently alleviating the severity of the disease of EAE[143]. Neural progenitor cell (NPC) transplant has been thoroughly investigated as an option for therapy for MS. NPCs produce leukemia inhibitory factor (LIF), which can upregulate SOCS3 expression and suppress Th17 cell-driven EAE[144]. Together, these results indicate that targeting SOCS3 may serve as a promising approach to MS treatment.

### Inflammatory bowel disease

Inflammatory bowel diseases (IBDs), which consist of Crohn’s disease (CD) and ulcerative colitis (UC), are chronic inflammatory disorders influencing the gastrointestinal tract, resulting in discomfort in the abdomen, persistent diarrhea, bowel bleeding, and loss of weight, and are linked to a heightened risk of colorectal cancer (CRC)[145]. Mucosal tissue of lesions in both UC and CD patients showed increased expression of SOCS1[146]. Dextran sulfate sodium (DSS) treated SOCS1^+/-^ mice showed deeper inflammation and more pronounced damage of the mucosa glandular architecture in the colon than DSS-treated WT mice[147]. This is due to the SOCS1 deficiency resulting in enhanced STAT1 activation and expression[147]. Thus, SOCS1 inhibits the progression of DSS-induced colitis by limiting STAT1 signaling. Mice with SOCS1 deficiency in all cells except T cells and B cells (SOCS1^-/-^-Tg) were susceptible to colitis, with reduced CTLA-4 and TGF-β expression[146]. This is presumed to be the degradation of CTLA-4 due to SOCS1 expression in T cells, which decreases TGF-β production, leading to disease aggravation[146]. Additionally, SOCS1^-/-^-Tg mice spontaneously developed colorectal cancer at six months of age, with SOCS1-deficient macrophage exhibiting hyperactivated STAT1 and increased production of cyclooxygenase-2 (COX-2) and inducible nitric oxide synthase (iNOS) triggered by IFN-γ[148]. IFN-γ is crucial for colitis-associated colorectal cancer (CAC) development. Studies have shown that SOCS1^-/-^-Tg mice administered anti-IFN-γ antibody and IFN-γ^-/-^SOCS1^-/-^ mice prevented CAC, suggesting that SOCS1 prevents the development of CAC by inhibiting the IFN-γ/ STAT1 signaling[148].

SOCS3 expression was elevated in biopsy specimens from patients with active UC compared to healthy controls[149]. Meanwhile, F59D-JAB transgenic mice with point mutations in the structural domain of KIR domains leading to SOCS3 dysfunction exhibit more severe colitis after DSS induction[150]. Furthermore, mice with SOCS3 knock-out in myeloid cells (lysM^cre^SOCS3^fl/fl^) showed increased neutrophil infiltration into the lamina propria after DSS treatment, exacerbating disease severity[151]. Thus, SOCS3 is a negative regulator in a DSS-induced model of IBD. Protein arginine methyltransferase 2 (PRMT2) methylates arginine residues on histones, targeting SOCS3 promoters to inhibit transcription[152]. DSS mice injected intrarectally with PRMT2 overexpressing lentivirus show increased disease activity due to decreased SOCS3 expression, resulting in hyperactivation of NF-κB and MAPK[152]. SOCS3 expression was reduced in cancer areas of the patients during UC-CRC progression[149]. The aryl hydrocarbon receptor (AhR) is a transcription factor that promotes the production of IL-22, which is essential for the effective initiation of DNA damage-induced repair following azoxymethane (AOM) exposure by STAT3 phosphorylation[153]. It was demonstrated that mice deficient in AhR in intestinal cells (AhR^fl/fl^Villin^Cre^) promote the development of AOM/DSS-induced CAC[154]. This is due to AhR deficiency increased SOCS3 expression and attenuated the effect of IL-22/STAT3 signaling, leading to the accumulation of DNA lesions and CAC development[154]. In conclusion, SOCS3 can inhibit the development of IBD, but the role of SOCS3 in the development of IBD developing into CAC needs to be further explored.

Aging exacerbates colitis in humans and mice, associated with increased expression of CISH in aged mice colonic epithelia[155,156]. Unlike young CISH^ΔIEC^ mice, aging CISH-deficient (CISH^ΔIEC^)mice showed protection against DSS-induced colitis due to higher glutathione S-transferase (GST) gene expression, which scavenging oxidative free radicals[156]. Furthermore, in aging colonic epithelial CCD841 cells, the knockdown of CISH reduced the expression of oxidative stress-related genes, including NCF2 and CYBB, and pro-inflammatory cytokines IL-1β and IL-6[156]. Based on these findings, CISH may have increased inflammation in the gut associated with aging by increasing oxidative stress, offering insights for treating IBD in the elderly.

### Psoriasis

Psoriasis is a chronically autoinflammatory dermatological disorder distinguished by epidermis proliferation, heightened angiogenesis, and the infiltration of inflammatory cells within the skin[157]. SOCS1^-/-^STAT1^-/-^ mice have demonstrated that massive migration of inflammatory cells into the skin leads to psoriasis-like skin disease, but it was significantly reduced in SOCS1^+/+^STAT1^-/-^ mice[158]. This is attributed to SOCS1 maintaining CCR7 expression by inhibiting STAT6 activation to maintain naive cells in lymph nodes, reducing effector cell entry into peripheral tissues and ultimately alleviating psoriasis[158]. Interferon-α (IFN-α) has been implicated in the pathogenesis of psoriasis[159]. SOCS1 inhibits Lys-63 polyubiquitination of Tyk2, leading to decreased expression of IFNAR1 and reduced JAK/STAT activation, which weakens IFN-α response[159]. Madonna et al.[160] found that SOCS1 KIR structural domain mimics disrupted the phosphorylation of JAK2 and STAT1 and reduced ICAM-1 expression in IFN-γ activated keratinocytes, thereby impairing T-cell adhesion to keratinocytes and ultimately alleviating disease severity of psoriasis.

The dysregulation of cytokine signaling in the keratinocytes is enough to induce psoriasis[161]. Mice with SOCS3 deficiency in keratinocytes resulted in overactivation of the IL-6-STAT3 pathway, which promoted the proliferation of keratinocytes, leading to severe clinical phenotypes resembling psoriasis in these mice[161]. Psoriatic arthritis (PsA) is a continuous inflammation of the joints linked to psoriasis, characterized by joint inflammation and the gradual deterioration of articular cartilage and bone[162]. Studies have shown that tofacitinib, a drug of the JAK inhibitor, significantly induced SOCS3 expression and decreased pSTAT3 and pSTAT1 in synovial explant cultures, which supports a role for SOCS3 in the treatment strategy for PsA[163]. These findings suggest that SOCS protein protects against the detrimental effects of persistent inflammatory activation in psoriasis.

### Type 1 diabetes

Type 1 diabetes mellitus (T1D) is an organ-specific immune system disorder marked by T cell-mediated pancreatic beta cell death, leading to their total reliance on insulin for maintenance[164]. According to earlier research, SOCS1 controls CD8^+^ T cell development and function, which impacts the course of the T1D disease process[[165], [166], [167]]. For instance, SOCS1 is capable of protecting against viral-induced T1D by impairing the CTL ability to kill β cells through the downregulation of MHC class I molecules and Fas expression[165]. In addition, elevated levels of SOCS1 inhibited the IL-15 signal, thereby restricting the expansion of CD8^+^ T cells[166]. The study also found that SOCS1 can protect β cells from cytokine-induced cell death, including IFN-γ and TNF-α, by decreasing caspase-3, caspase-8, and caspase-9 activation[167]. It has been investigated that targeting SOCS2 by nanoparticles (NPs) is involved in the development of T1D[168]. NP_ITE+Ins_ is a type of NPs that can deliver a tolerogenic molecule, suppressing T1D development in non-obese diabetic (NOD) mice by inducing the generation of tolerogenic DCs[168]. NP_ITE+Ins_ administration enhanced AhR activation, which increases SOCS2 expression to inhibit NF-kB p65 activation and ultimately increases tolerogenic DC generation[168].

Studies have shown that SOCS3 alleviates T1D by inhibiting STAT3 activation and β-cell apoptosis[[169], [170], [171]]. Previous studies have shown that SOCS3 inhibits IL-1[170] and IFN-γ[169] signaling, protecting β cells from cytokines-induced cell apoptosis. Additionally, a study has shown that Gpr41^-/-^ mice exhibited markedly aggravated T1D progression following streptozotocin (STZ)-induction in comparison to WT mice[171]. G-protein-coupled receptor 41 (GPR41) is a short-chain fatty acid (SCFA) receptor, that maintains intestinal homeostasis. This is due to BM-derived DCs from Gpr41^-/-^ mice exhibiting decreased SOCS3 production and increased STAT3 activation, suggesting SOCS3 is involved in T1D development[171].

All of the above findings summed up the protective function of SOCS protein in T1D pathogenesis and helped to promote the advancement of T1D treatments.

### Sjögren's syndrome

Primary Sjögren's syndrome (pSS) is a prototypic autoimmune syndrome marked by lymphocytic infiltration of salivary and lachrymal glands, which results in the sicca symptoms, primarily dry mouth and eye dryness[172]. A study has found that SOCS3 expression was highest in SS patients, intermediate in patients with sicca syndrome, who exhibit sicca symptoms without meeting diagnostic criteria for SS, and lowest in healthy controls[173]. The results indicate that the expression level of SOCS correlates with disease severity. A study has shown a notable prolongation of STAT3 phosphorylation in IL-6-treated peripheral blood mononuclear cells (PBMC) from SS patients[173]. This may be due to a functional dysfunction of the signaling cascade between SOCS3 and pSTAT3 in SS patients or the failure of SS patients to produce more SOCS3 to inhibit pSTAT3 expression, requiring further investigation into the mechanism.

### Atherosclerosis

Atherosclerosis is characterized by the infiltration of inflammatory cells and the accumulation of oxidized lipoproteins within the arterial walls of large and medium-sized arteries[174]. Research has increasingly indicated that atherosclerosis shares similar hallmark immunological features with autoimmune diseases, including self-antigen recognition, T cell-driven inflammation, and tolerance breakdown[175]. It was shown that enhancing SOCS1 and SOCS3 expression by adenovirus inhibits vascular STAT1/STAT3 activation suppressing inflammation and the development of atherosclerosis in mice[176]. Furthermore, the peptidomimetic of KIR-SOCS1, PS5, was demonstrated to suppress NADPH oxidase and the expression of pro-inflammatory genes, thereby decreasing atheroma plaque size and lipid content[177,178]. In summary, SOCS proteins decelerate the process of atherosclerosis by inhibiting pro-inflammatory signaling and maintaining the balance of immune tolerance, and targeting SOCS proteins provides a new direction for immunotherapy to intervene in the transformation of atherosclerosis to autoimmune features.

### Cancers with chronic inflammation

While the present review focuses on the regulatory role of SOCS proteins in autoimmune diseases, it should be noted that their potential immunomodulatory role is in a broader pathophysiological context, particularly in the cancer-associated inflammatory microenvironment. In addition to its function in regulating JAK/STAT signaling, SOCS 1 also plays a pivotal role in the suppression of NF-κB transcription through the ubiquitination and subsequent degradation of the NF-κB p65 subunit in breast cancer[55]. It has been shown that the JAK2-targeting PEGylated SOCS3 mimetic therapeutically suppresses triple-negative breast cancer progression through MDA-MB-231 cell penetration and STAT3 phosphoregulation[179]. While extant reviews comprehensively map SOCS involvement in tumorigenesis, mechanistic delineation of their context-dependent regulatory dynamics at the chronic inflammatory priming-oncogenic transformation interface remains an imperative research frontier[180].

SOCS family affects the development and function of innate and adaptive immune cells, with abnormal SOCS expression associated with autoimmune diseases.

## Concluding remarks and future perspectives

Current immunosuppressive therapies for autoimmune diseases (e.g., corticosteroids, TNF-α/IL-6-targeting biologics) remain limited by poor patient responsiveness and infection/metabolic risks, driving exploration of advanced approaches like mesenchymal stem cell-derived exosomes (MSC-Exos) and CAR-T cell therapy[181]. In the decades since the identification of the SOCS family, numerous studies on the structure and function of SOCS molecules have identified them as crucial cellular inhibitors of cytokine signaling pathways. The latest evidence highlights the vital significance of SOCS proteins in the underlying cause of various autoimmune disorders. Several SOCS-related mimetic peptides have been studied in preclinical studies. One approach is through the injection of adenoviral vectors overexpressing SOCS proteins. For example, intra-articular injection (IA) of recombinant adenovirus expressing SOCS3 reduced joint inflammation and prevented the development of CIA[118]. An alternative strategy involves using molecular mimics of different SOCS molecules. SOCS mimics are emerging as innovative therapeutic alternatives for autoimmune diseases due to their compact size, stability, and minimal immunogenicity, making them strong candidates for safe treatment development. The SOCS1 mimetic tyrosine kinase inhibitory peptide (Tkip) was produced utilizing hydropathic similarity to the JAK2 activation loop, inhibited transcription factor STAT1/JAK2 phosphorylation, thereby alleviating disease severity in EAE mice[134]. Additionally, SOCS1 KIR structural domain mimics disrupted the phosphorylation of STAT1/JAK2, thereby alleviating disease severity in psoriasis[160]. Different from Tkip, SOCS1-KIR is unable to inhibit JAK2 phosphorylation[182].

Currently, most studies on the SOCS family regulation have concentrated on experimental animals and preclinical studies, so deeper clinical investigations on the particular functions of SOCS molecules in different autoimmune diseases must be validated. (The information comes from clinicaltrials.gov.) CRISPR/Cas9 knockouts of CISH in tumor-infiltrating lymphocytes (TILs) increased TIL function and increased the sensitivity of PD-1 blockade, ultimately increasing anti-tumour activity [183]. This finding established the theoretical basis for a clinical trial (NCT04426669) in testing CISH knockout TILs in patients with metastatic gastrointestinal cancers. It is crucial to conduct research and clinical trials to thoroughly understand their mechanisms, safety, and efficacy across various autoimmune disorders before they can be widely adopted in medical practice. Further investigations will enhance comprehension of the significance of SOCS in immune dysregulations and autoimmune pathogenesis, which may contribute to discovering innovative targets for treating autoimmune diseases.

## Author contributions

**Liwei Lu:** Conceptualization, writing-review & editing. **Ke Rui:** Funding acquisition, writing-review&editing. **Jie Tian:** Funding acquisition, writing-review&editing. **Dongmei Zhou:** Writing-review&editing. **Mingwei Wang:** Writing-review&editing. **Liangjie Xu:** Writing-review & editing. **Daihua Deng:** Writing-review&editing. **Shiyi Liu:** Writing-review&editing.

## Funding

This work was supported by the National Natural Science Foundation of China (Grant Nos. 82171771, 82271854, 82300283), the Jiangsu Provincial Key Research and Development Program (Grant number BE2023758), Major Project of Basic Science Research in Higher Education Institutions of Jiangsu Province (23KJA320001), Jiangsu Association for Science and Technology's Young Talents Nurturing Program (TJ-2023–027), the Science and Technology Support Program (Social Development) of Zhenjiang (SH2023088).

## Declaration of competing interest

*The authors declare that they have no known competing financial interests or personal relationships that could have appeared to influence the work reported in this paper.*
